# Ultrasound and MRI findings as predictors of propranolol therapy response in patients with infantile hemangioma

**DOI:** 10.1371/journal.pone.0247505

**Published:** 2021-03-10

**Authors:** Hee Jin Park, So-Yeon Lee, Myung Ho Rho, Hye Lim Jung

**Affiliations:** 1 Department of Radiology, Kangbuk Samsung Hospital, Sungkyunkwan University School of Medicine, Seoul, Republic of Korea; 2 Department of Radiology, Seoul St. Mary’s Hospital, School of Medicine, The Catholic University of Korea, Seoul, Republic of Korea; 3 Department of Pediatrics, Kangbuk Samsung Hospital, Sungkyunkwan University School of Medicine, Seoul, Republic of Korea; National Institutes of Health, UNITED STATES

## Abstract

**Objectives:**

To evaluate the prognostic value of ultrasound and MRI findings in patients with infantile hemangioma undergoing propranolol therapy.

**Methods:**

This study was based on retrospective interpretation of prospectively acquired data. Thirty-eight consecutive patients (28 females and 10 males; mean age ± standard deviation, 3.2 ± 2.2 months) who underwent propranolol treatment for infantile hemangioma were included. Pre-treatment ultrasound images were assessed in terms of echogenicity, lesion height and vascularity. Presence of prominent intratumoral fat, non-fat septa, and enhancement pattern on MRI were retrospectively evaluated. Mann-Whitney test, chi-square, and Fisher’s exact tests were used to compare imaging parameters between patients with treatment success and failure.

**Results:**

All patients underwent ultrasound and 15 patients underwent MRI. A total of 24 patients showed successful treatment. Between patients with treatment success and failure, there were significant differences in increased vascularity on pre-treatment ultrasound (19/24 vs. 6/14, p = 0.025), decreased vascularity on post-treatment ultrasound (21/24 vs. 5/14, p = 0.001), and prominent intratumoral fat on MRI (1/8 vs. 5/7 p = 0.033). There were no significant differences in echogenicity, lesion height on ultrasound, non-fat septa and MR enhancement pattern.

**Conclusions:**

Increased vascularity on pre-treatment ultrasound was significantly associated with successful treatment for propranolol therapy in patients with infantile hemangioma, whereas prominent fat component on MRI was significantly associated with treatment failure.

## Introduction

Infantile hemangioma is the most common neoplasm of infants and young children [1]. Infantile hemangiomas usually develop during the first 4 to 6 weeks of life, and most growth occurs during the first 5 months [2]. Although most lesions show spontaneous regression and uncomplicated clinical course, some lesions leave sequelae or complications such as permanent disfigurement, ulceration, bleeding, or airway involvement [3]. Approximately 20% of infantile hemangiomas have complications and some of them require treatment [4].

Propranolol, a nonselective β-blocker, has been adopted as a first-line therapy for infantile hemangiomas requiring systemic therapy over the past few years [5–9]. Side effects of propranolol include transient bradycardia, hypotension, agitation, bronchospasm and hypoglycemia [7–10]. Other treatment options for infantile hemangioma include steroid, interferon alpha, vincristine, and laser treatment [11–13]. The efficacy of these treatments varies and each treatment option has associated safety concerns. Propranolol has been effective in reducing the size of infantile hemangioma; some patients show partial regression rather than complete regression [6, 7]. Propranolol treatment response prediction helps establish treatment plans, considering the side effects and other treatment options.

Ultrasound (US) and magnetic resonance imaging (MRI) are used for detection, diagnosis and monitoring of treatment response for infantile hemangioma [7, 14]. Hypervascularity seen as abundant blood flow signals on US Doppler imaging and strong enhancement on MRI is the typical imaging finding of infantile hemangioma on proliferative phase. Considering the anti-angiogenesis effect of propranolol, we hypothesized that US and MRI findings can be used to predict treatment response. The correlation between pre-treatment imaging findings and treatment response is not well known in patients with infantile hemangioma. Therefore, the purpose of this study was to explore imaging predictors of successful treatment with propranolol therapy in patients with infantile hemangioma.

## Materials and methods

This study was based on retrospective interpretation of prospectively acquired data. The Kangbuk Samsung Hospital Institutional Review Board (IRB) approved this study, and written informed consent was waived.

### Study population

**Fig 1:**
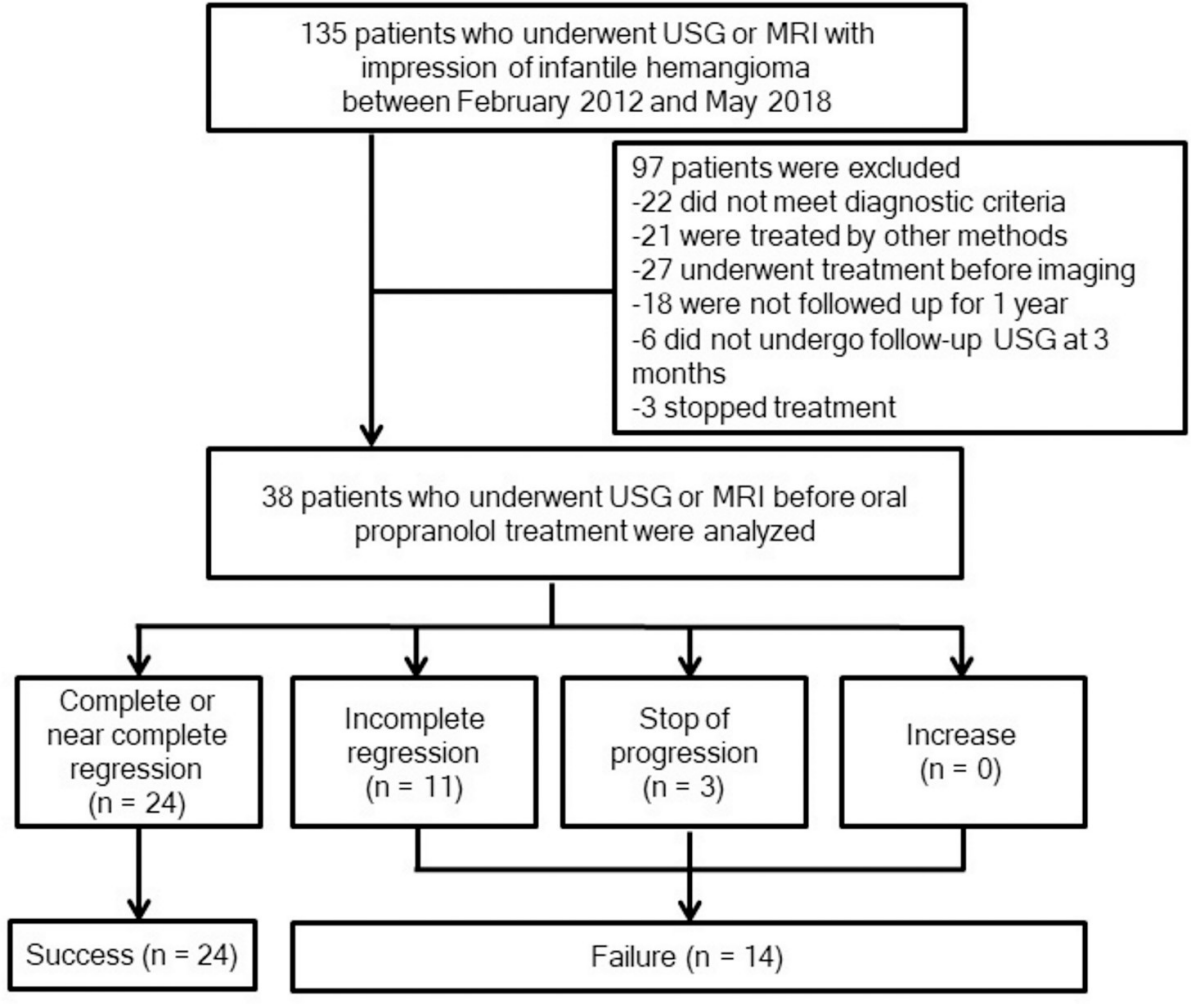
Flow diagram for inclusion of study subjects.

We retrospectively reviewed data from all patients with infantile hemangioma who visited our infantile hemangioma clinic between February 2012 and May 2018. One experienced pediatric physician (H.L.J., with 29 years of experience in pediatric hematology and oncology) treated all patients with the following clinical treatment and monitoring protocol [15]. US was performed before treatment and within 1–3 months of treatment. Patients with hemangioma involving any tissue except other than skin and subcutaneous fat (i.e., lip, salivary gland, muscle) underwent MRI before treatment. Propranolol was administered at a dosage of 2 mg per kilogram per day divided into three daily doses continuing for duration of at least 6 months [8, 9]. Propranolol doses were adjusted as follows: 0.5 mg per kilogram per day on day 0, 1.0 mg per kilogram per day on day 1, and 2.0 mg per kilogram per day on day 2. A total of 135 eligible patients were identified from medical records. The inclusion criteria were patients with newly diagnosed infantile hemangioma treated with oral propranolol in our hospital, patients who have undergone at least one US or MRI for infantile hemangioma, and patients who have been followed up for at least one year. Exclusion criteria were patients who had undergone any prior hemangioma treatment, patients who had tried combined therapy during a 1-year follow-up period and patients with presumed infantile hemangioma who did not meet the diagnostic criteria of infantile hemangioma upon retrospective review of clinical and imaging findings [16–18]. Patients who were older than 9 months were also excluded [7]. In addition, patients without US in the first three months of treatment were excluded. The final study population consisted of 28 females (mean age ± standard deviation, 3.5 ± 2.3 months) and 10 males (3.1 ± 2.1 months) (Fig 1). Fifteen patients underwent pretreatment MRI among the final study population.

### Clinical characteristics and treatment outcome

The clinical characteristics of patients with infantile hemangioma were obtained from medical records including regular follow-up pictures of the lesion; data included gender, age at initial visit, multiplicity, location, deep tissue involvement, and size of hemangiomas. The largest lesion was selected when a patient presented multiple hemangiomas. Deep tissue involvement was defined as involving any tissue other than skin and subcutaneous fat (i.e., muscle, salivary gland) by visual inspection and imaging findings, and was classified as presence or absence. Treatment outcome was assessed at 1-year follow-up according to the obtained pictures and medical imaging of the lesion at baseline and posttreatment. Outcomes were classified as follows according to previously reported literatures by one experienced pediatric physician (H.L.J.) [6, 7]: *complete or near complete regression* was defined as complete regression of the target infantile hemangioma without sequelae or with minimal sequelae. Minimal sequelae were defined as minimal telangiectasias, macular discoloration, and/or textural change. *Incomplete regression* was defined as volume decrease by 25% or more compared to the original size. *Stop of progression* was defined when the volume did not increase or decrease by less than 25% after treatment. An *increase* was defined if the volume measured at evaluation was greater than the size measured at beginning of treatment. Complete regression was defined as treatment success. Incomplete regression, stop of progression, and increase were defined as treatment failure.

### US findings

Two musculoskeletal radiologists (H.J.P. and S.Y.L., with 18 and 10 years of experience in musculoskeletal radiology, respectively) performed all ultrasonographic examinations using HDI 5000 (Philips Medical Systems, Bothell, Washington) and Logiq E9 (GE Healthcare, Milwaukee, Wisconsin) imaging devices equipped with linear 6- to 15-MHz probes. Gray-scale images and color Doppler images were obtained for infantile hemangiomas in transverse and longitudinal planes. At least one image was obtained at the dimension showing the maximum diameter of the lesion. Standardized parameter settings were used for Doppler exam as follows: pulse repetition frequency between 700–1000 Hz, velocity scale between 7cm/sec to -7cm/sec, low wall filter (50Hz), maximum gain (85%–90%), medium persistence, and box without angulation.

**Fig 2:**
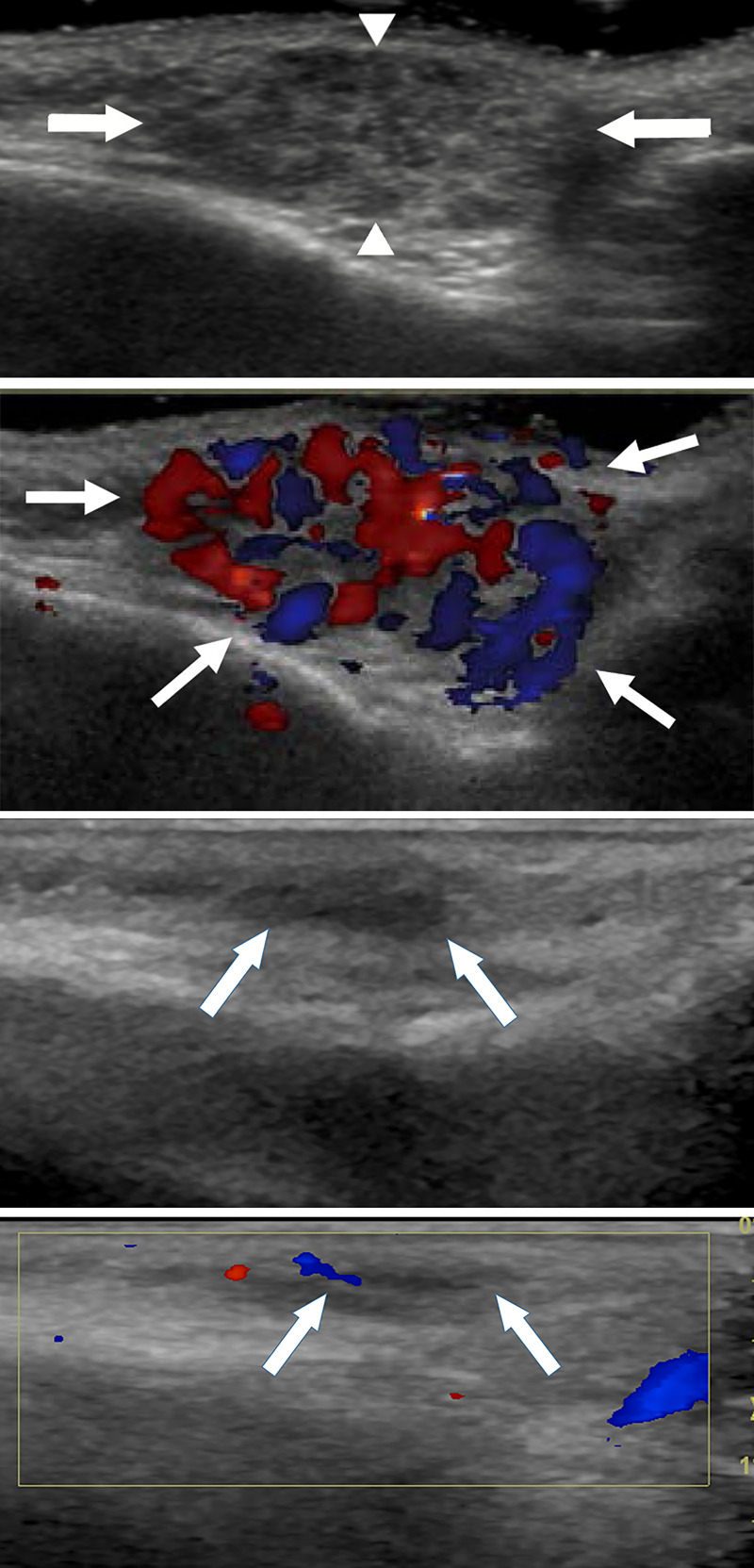
Image of a 4-month-old girl with infantile hemangioma who had near complete regression after 1 year of propranolol treatment. (a) Initial gray-scale US image shows a hypoechoic subcutaneous mass (arrows) without deep tissue involvement. Lesion length was defined as the longest diameter of the tumor (distance between arrows). Lesion height was defined as the longest distance between superficial and deep margins of the lesion (distance between arrowheads). (b) Initial color Doppler image depicts hypervascularity within the lesion (arrows). (c) Follow-up gray-scale US image shows decreased size of the lesion (arrows) (d) Follow-up color Doppler image depicts hypovascularity within the lesion (arrows), showing interval decreased vascularity.

US imaging results were retrospectively reviewed with consensus from both experienced radiologists (H.J.P. and S.Y.L.). Discordances between readers were handled according to a third radiologist’s decision (M.H.R., with 26 years of experience in neuroradiology). Both radiologists were blinded to imaging reports, age, gender, and treatment results for evaluation of US imaging. US findings were evaluated according to echogenicity, lesion length, height, and vascularity (Fig 2). The internal echotexture of each lesion was classified as hyperechoic, mixed, and hypoechoic. When echogenicity was hyperechoic or hypoechoic to subcutaneous fat in more than half of the lesion volume, it was classified as hyperechoic or hypoechoic, respectively. Other lesions were classified as mixed echogenicity. Lesion length was defined as the longest diameter of the tumor. Lesion height was defined as the longest distance between superficial and deep margins of the lesion. Both lesion length and height were measured on a picture archiving and communication system (PACS) system. Vascularity was evaluated on color Doppler imaging according to binary scale: hypovascularity and hypervascularity. The most abundant blood flow signals on a single plane was selected as the defined tumor section for visual assessment. Hypovascularity was defined as blood flow signal in less than 50% of the tumor area (Fig 2D) and hypervascularity was defined as blood flow signal in more than 50% of the tumor area (Fig 2B) [19, 20].

By comparing the initial and follow-up US, the size reduction rate for each lesion height and length was calculated as follows. The difference was the value obtained by subtracting the lesion height/length measured at follow-up US from the lesion height/length measured at initial US. Size reduction rate was calculated as a percentage by dividing the difference value by the lesion height/length value measured at initial US and multiplying it by 100. Initial Doppler US and follow-up Doppler US were compared to evaluate the change in vascularity. When the blood flow signal changed from hypervascularity to hypovascularity or the blood flow signal decreased by more than half on the follow-up US compared to the initial US, it was defined as decreased vascularity.

### MRI findings

MRI was acquired with a 3.0 T MRI system (Achieva, Philips Healthcare) and dedicated surface coils for anatomic location. Images were obtained in axial, coronal, and sagittal planes. T1-weighted images, T2-weighted images, fat-suppressed T2-weighted images, and T1-weighted fat-suppressed contrast-enhanced MR images were obtained for at least one plane. MRI parameters were as follows: field-of-view (FOV) 160 x 160–180 x 180, repetition time/echo time (TR/TE) for T1- (650-872/10) and T2- (3387-4643/70-80) weighted images, number of excitations (NEX) 2–6, matrix 204–268 x 193–198, and thickness/gap 3/0.3–5/6.

**Fig 3:**
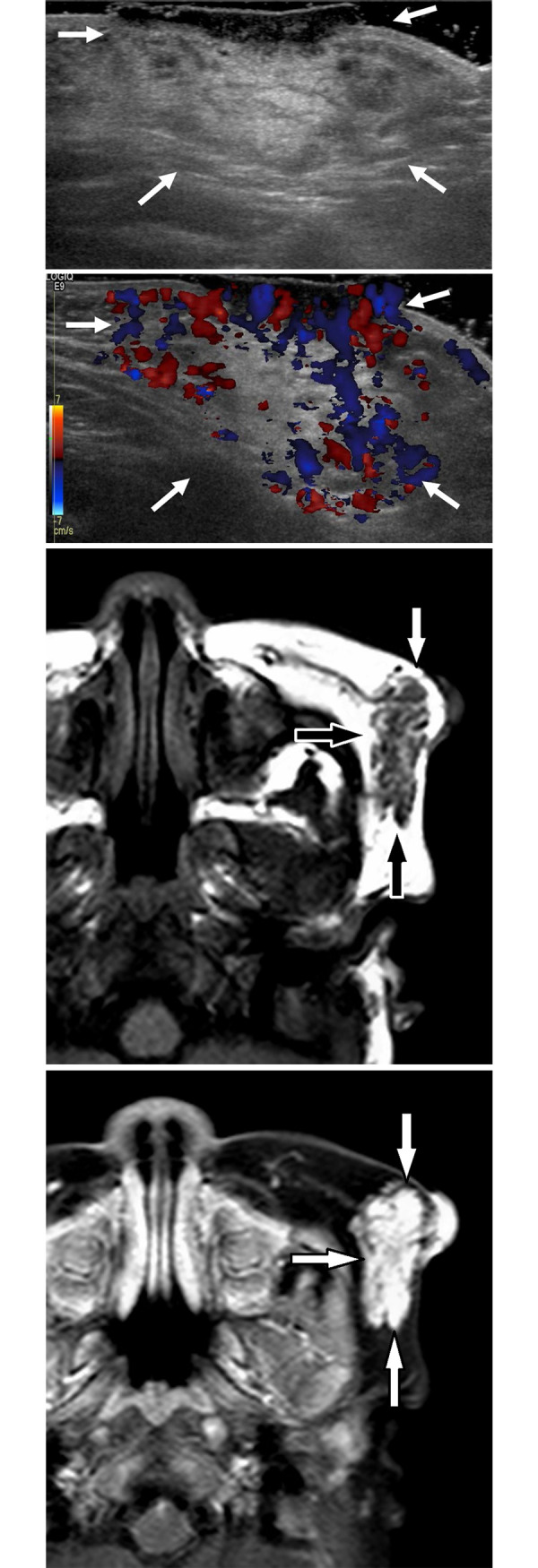
Image from a 4-month-old girl with infantile hemangioma who showed incomplete regression after 1 year of propranolol treatment. (a) Gray-scale US image shows a hyperechoic subcutaneous mass with a thick skin lesion (arrows). (b) Color Doppler image reveals hypo vascularity within the lesion (arrows). (c) Axial T1-weighted MRI of the face shows a lobulated mass (arrows) with multiple fatty septa. (d) Axial T1-weighted fat-suppressed contrast-enhanced MR image of the face reveals homogeneously strong enhancement (arrows).

MRI images were retrospectively reviewed with consensus between two radiologists (H.J.P. and S.Y.L). Discordances between the two readers were resolved according to a third radiologist’s decision (M.H.R.). Pretreatment MRI findings were evaluated according to fat component, non-fat septa, and enhancement (Fig 3). Prominent fat component was classified as present or absent. Minimal fat, which appeared as unmeasurable tiny spots, was classified as absent. Septa-like nodular fat components were classified as present. Non-fatty septa components were defined as linear portions without fat attenuation and were classified as present or absent. Enhancement was classified as homogeneous or heterogeneous enhancement.

### Statistical analysis

Analysis was performed with SPSS Statistics software (version 24; IBM, Armonk, NY). Clinical and imaging parameters were compared between treatment success and failure groups using the Mann-Whitney U test for continuous variables and the chi-square test or Fisher exact test for proportions. P value of 0.05 was used as the cutoff for statistical significance.

## Results

### Baseline clinical characteristics

A total of 24 patients showed complete (n = 12) or near complete regression (n = 12), 11 patients showed incomplete regression, and 3 patients showed stop of progression. No patient showed increased size of hemangioma (Table 1). Median age at inclusion was 3.0 months (interquartile range, 2.0 to 4.0 months). Median length of infantile hemangioma was 2.8cm (interquartile range, 1.6 to 4.5 cm).

**Table 1:** Baseline clinical characteristics.

Variable	Patients (n = 38)
**Clinical findings**	
**Gender**	
** Male**	10
** Female**	28
**Age at inclusion (mo)**	
** ≤ 5 mo**	33
** >5 mo ≤ 9 mo**	5
**Infantile hemangioma**	
**Multiplicity**	
** Multiple**	7
** Single**	31
**Location**	
** Head and neck**	25
** Others**	13
**Deep tissue involvement**	
** Present**	8
** Absent**	30
**Treatment response**	
** Complete or near complete regression**	24
** Incomplete regression**	11
** Stop of progression**	3
** Increase**	0

Values are the number of patients.

### Clinical and imaging characteristics according to successful treatment

All clinical parameters including gender, age, multiplicity, location, deep tissue involvement, and size of infantile hemangioma were not significantly different between treatment success and failure groups (p = 0.081 to 0.883, Table 2). Increased vascularity on pre-treatment US was significantly more frequent in patients with successful treatment (19/24 vs. 6/14, p = 0.025, Table 3). No significant differences were noted in echogenicity, and lesion height on US (p = 0. 466 and 0.868, respectively). Prominent fat component was more often observed on pre-treatment MRI from patients with treatment failure (5/7 vs. 1/8 p = 0.033, Table 4). Non-fatty septa, deep tissue involvement, and enhancement pattern on MRI were not significantly different (p = 0.077 to 0.922). Decreased vascularity on follow-up US imaging at 3-month follow-up compared to pre-treatment imaging was significantly frequent in successful treatment group compared to failure group (21/24 vs. 5/14, p = 0.001, Table 5). Increased echogenic portion of the lesion and size reduction were not significantly different between two groups (p = 0.056 to 0.310).

**Table 2:** Clinical characteristics according to successful treatment.

Variable	Success group (n = 24)	Failure group (n = 14)	P-value
**Patient**			
**Gender**			0.081
** Male**	4	6	
** Female**	20	8	
**Age at inclusion (mo)**	2.5 (2.0 to 4.0)	3.0 (1.0 to 4.0)	0.701
**Hemangioma**			
**Multiplicity**			0.177
** Multiple**	6	1	
** Single**	18	13	
**Location**			0.883
** Facial**	16	9	
** Non-facial**	8	5	
**Deep tissue involvement**			0.113
** Present**	7	1	
** Absent**	17	13	
**Size (cm)**	3.00 (1.55 to 4.25)	2.45 (1.60 to 5.40)	0.856

Values are number of patients or median (interquartile range).

Mann-Whitney test, Chi-test, and Fisher’s exact test were performed.

**Table 3:** Pre-treatment ultrasonographic imaging findings according to successful treatment.

Variable	Success Group (n = 24)	Failure group (n = 14)	P-value
**Echogenicity**			0.466
** Hypoechoic**	13	6	
** Mixed**	8	4	
** Hyperechoic**	3	4	
**Lesion size (cm)**			
** Length**	2.80 (1.80 to 3.65)	2.15 (1.70 to 4.00)	0.716
** Height**	0.75 (0.40 to 0.95)	0.65 (0.40 to 1.30)	0.868
**Vascularity**			0.025*
** Hypervascular**	19	6	
** Hypovascular**	5	8	

Values are number of patients or median (interquartile range).

Mann-Whitney test, Chi-test, and Fisher’s exact test were performed.

*, Statistically significant.

**Table 4:** Pre-treatment MR imaging findings according to successful treatment.

Variable	Success Group (n = 8)	Failure group (n = 7)	P-value
**Prominent fat component**			0.033*
** Present**	1	5	
** Absent**	7	2	
**Non-fatty septa**			0.838
** Present**	3	5	
** Absent**	5	2	
**Enhancement**			0.922
** Homogeneous**	7	6	
** Heterogeneous**	1	1	

Values are number of patients or median (interquartile range).

Mann-Whitney test, Chi-test, and Fisher’s exact test were performed.

*, Statistically significant.

**Table 5:** Follow-up ultrasonographic imaging findings at 3 months after starting treatment.

Variable	Success group (n = 24)	Failure group (n = 14)	P-value
**Increased hyperechoic portion**	9	3	0.310
**Size reduction rate (%)**			
** Length**	20.0 (3.1 to 44.7)	9.2 (0.0 to 26.1)	0.136
** Height**	37.5 (5.3 to 54.7)	14.3 (0 to 37.5)	0.056
**Decreased vascularity**	21	5	0.001*

Values are number of patients or median (interquartile range).

Mann-Whitney test, Chi-test, and Fisher’s exact test were performed.

*, Statistically significant.

## Discussion

Hypervascularity on pre-treatment color Doppler imaging and decreased vascularity on early follow-up imaging during treatment were prognostic factors of successful treatment with propranolol therapy. These results suggest that the more the vascular burden of infantile hemangioma increases, the more the infantile hemangioma is responsive to propranolol. These may be a natural result because propranolol induces vasoconstriction and anti-angiogenetic effects [21]. Doppler exam effectively showed the decreased vascular burden of infantile hemangiomas regarding treatment. This finding is consistent with results from previous studies. Babiak-Choroszczak et al. [22] showed significant decrease in vascular endothelial growth factor (VEGF) and basic fibroblast growth factor (bFGF), and decreased blood flow in Doppler US during and after propranolol treatment. According to Shi et al. [23], Doppler parameters including vascular density, blood flow velocity, and arterial peak systolic blood flow velocity significantly decreased in infantile heamangiomas after propranolol treatment.

Prominent fat component on MRI was associated with treatment failure for propranolol therapy in patients with infantile hemangioma. MRI can be used to distinguish fibrotic tissue from fatty tissue in infantile hemangiomas because fat tissue is seen as hyperintense signal on T1- and T2-weighted images with hypointensity on fat-suppressed images. US cannot discriminate fibrotic tissue and fatty tissue because both are hyperechogenic. This may be the reason why increased fatty tissue on MRI correlated with poor prognosis in our study, whereas the hyperechoic portion did not correlate with poor prognosis. The mechanism of association between intratumoral fat and poor response for propranolol is not clear.

In general, the amount of fat in infantile hemangioma increases with age according to natural evolution. The imaging findings of infantile hemangiomas depend on biologic phase [16, 17]. In the proliferative phase, infantile hemangioma shows as well circumscribed mass of variable echogenicity with high vascularity on US; it appears as well-defined mass with high signal intensity on T2-weighted images and with intermediate signal intensity on T1-weighted images. Flow voids can be visible on spin-echo images. After injection of gadolinium contrast material, early intense and uniform enhancement is observed. During the involuting phase, there is an increased echogenicity on US and decreased vascularity as a result of fibro-fatty replacement. Involuted infantile hemangioma shows increased amount of fat seen as hyperintensity on T1-and T2-weighted images and less avid enhancement after contrast material injection. We found no significant difference in age at the start of therapy between the treatment success and failure groups. This finding is consistent with results from previous studies [24, 25]. According to a multicenter retrospective study, propranolol therapy was effective in infantile hemangiomas in the post-proliferative phase as well as in the proliferative phase [24]. According to another study from Schupp et al. [25], therapy started at an older age results in clinical improvements, although it is less effective than in very young children.

Diagnosis is the primary role of imaging in patients with infantile hemangioma. It is optionally recommended when the diagnosis is uncertain, more than five superficial infantile hemangiomas are present, or if anatomical problems exist [25, 26]. The initial imaging modality of choice is US, and MRI can be used for evaluation of anatomical structures [26]. We have newly discovered that initial and early follow-up imaging findings are related to treatment results. Whether imaging should be performed for the purpose of predicting therapeutic effect of propranolol on infantile hemangioma is outside the scope of our study. The results of this study are that evaluating blood flow and fat components in infantile hemangioma imaging performed by clinical needs can provide additional predictive information on treatment response. Oral propanol is the first-line agent for infantile hemangiomas requiring systemic treatment. Prednisolone may be used if there is any contraindication or an inadequate response to oral propranolol. Intratumoral injection of triamcinolone and/or betamethasone, surgery or laser therapy can be combined. In these situations, prediction of treatment responses to oral propranolol may be helpful in managing infantile hemangioma. However, there have been few reports regarding prediction of responses to propranolol in patients with infantile hemangioma [27]. A recent multicenter study found that patients with higher serum bFGF and VEGF levels showed better response to propranolol at 1-year follow-up. Imaging may provide additional prognostic information by a noninvasive method.

This study has some limitations. First, severe infantile hemangiomas were not included because these patients were first treated with combined therapy of propranolol and other medications. Most of these patients were referred because they did not respond well to treatment at other hospitals. Second, this was a retrospective study and patients who were lost during follow-up were not included. Finally, only some patients underwent MRI.

In conclusion, high vascularity on pre-treatment color Doppler US and decreased vascularity on early follow-up US imaging during treatment were predictors of successful treatment of infantile hemangioma with propranolol therapy. Prominent fatty tissue on MRI was significantly associated with poor prognosis.

## Supporting information

S1 File (XLSX)
